# Identification of a novel gene signature of lung adenocarcinoma based on epidermal growth factor receptor-tyrosine kinase inhibitor resistance

**DOI:** 10.3389/fonc.2022.1008283

**Published:** 2022-12-01

**Authors:** E. Zhou, Feng Wu, Mengfei Guo, Zhengrong Yin, Yumei Li, Minglei Li, Hui Xia, Jingjing Deng, Guanghai Yang, Yang Jin

**Affiliations:** ^1^ Department of Respiratory and Critical Care Medicine, Hubei Province Clinical Research Center for Major Respiratory Diseases, Key Laboratory of Pulmonary Diseases of National Health Commission, Union Hospital, Tongji Medical College, Huazhong University of Science and Technology, Wuhan, China; ^2^ Hubei Province Engineering Research Center for Tumor-Targeted Biochemotherapy, Key Laboratory of Biological Targeted Therapy, the Ministry of Education, Union Hospital, Tongji Medical College, Huazhong University of Science and Technology, Wuhan, China; ^3^ Department of Thoracic Surgery, Union Hospital, Tongji Medical College, Huazhong University of Science and Technology, Wuhan, China

**Keywords:** prognostic signature, lung adenocarcinoma, epidermal growth factor receptor-tyrosine kinase inhibitor resistance, immune microenvironment, drug sensitivity

## Abstract

**Introduction:**

Tyrosine kinase inhibitors (TKIs) that target epidermal growth factor receptor (EGFR) mutations are commonly administered to EGFR-positive lung cancer patients. However, resistance to EGFR-TKIs (mostly gefitinib and erlotinib) is presently a significant problem. Limited studies have focused on an EGFR-TKI resistance-related gene signature (ERS) in lung adenocarcinoma (LUAD).

**Methods:**

Gefitinib and erlotinib resistance-related genes were obtained through the differential analyses of three Gene Expression Omnibus datasets. These genes were investigated further in LUAD patients from The Cancer Genome Atlas (TCGA). Patients in the TCGA-LUAD cohort were split into two groups: one for training and one for testing. The training cohort was used to build the ERS, and the testing cohort was used to test it. GO and KEGG analyses were explored for the enriched pathways between the high-risk and low-risk groups. Various software, mainly CIBERSORT and ssGSEA, were used for immune infiltration profiles. Somatic mutation and drug sensitivity analyses were also explored.

**Results:**

An ERS based on five genes (FGD3, PCDH7, DEPDC1B, SATB2, and S100P) was constructed and validated using the TCGA-LUAD cohort, resulting in the significant stratification of LUAD patients into high-risk and low-risk groups. Multivariable Cox analyses confirmed that ERS had an independent prognostic value in LUAD. The pathway enrichment analyses showed that most of the genes that were different between the two risk groups were related to the immune system. Further immune infiltration results revealed that a lower immune infiltration score was observed in high-risk patients, and that various leukocytes were significantly related to the ERS. Importantly, samples from the high-risk group showed lower levels of PD-1, PD-L1, and CTLA-4, which are important biomarkers for immunotherapy responses. Patients in the high-risk group also had more gene mutation changes and were more sensitive to chemotherapy drugs like docetaxel and sorafenib. The ERS was also validated in the GSE30219, GSE11969 and GSE72094, and showed a favorable prognostic value for LUAD patients.

**Discussion:**

The ERS established during this study was able to predict a poor prognosis for LUAD patients and had great potential for predicting drug responses.

## Introduction

Lung cancer is one of the most common and deadly types of cancer (1). Non-small-cell lung cancer (NSCLC), which makes up 85% of lung cancer cases, is thought to be the most common type (2). Adenocarcinoma, squamous cell carcinoma, and large cell carcinoma are the three main subtypes of NSCLC (2). Among these histological phenotypes, adenocarcinomas of the lung (LUAD) is the most prevalent one. Even though lung cancer screening and systemic treatments have improved, most patients don’t respond well to treatment, and the 5-year survival rate for lung cancer patients is only 4% to 17% (3). Molecular technology and targeted therapies can extend the overall survival (OS) of lung cancer patients (4, 5), but resistance to targeted drugs is still a big problem.

The epidermal growth factor receptor (EGFR) gene is often changed in people with NSCLC. About 10% to 20% of Caucasian patients and about 50% of Asian patients have EGFR gene mutations (6, 7). In lung cancer patients with EGFR gene mutations, tyrosine kinase inhibitors (TKIs) that target mutant EGFR genes are associated with a therapeutic advantage. As an established first-line standard therapy, EGFR-TKIs have greater efficacy than standard chemotherapy (8). However, because of individual mutations and varieties of EGFR-TKIs, patients have diverse sensitivity to EGFR-TKIs and will eventually experience disease progression because of acquired resistance (8, 9). First-generation EGFR-TKIs, primarily gefitinib and erlotinib, are the most frequently used and prevalent treatments for acquired resistance (10). Various trials in clinic have implied that second-generation and third-generation TKIs are more effective than first-generation TKIs (11–13). Although the second-generation and third-generation EGFR-TKIs are substitutes for first-generation EGFR-TKIs, drug resistance and poor LUAD prognoses are problematic. Therefore, new biomarkers are necessary for predicting better prognoses.

With the development of bioinformatics analyses, it has become convenient to use public genomic datasets for risk model construction and survival prediction. In our study, we explored the potential value of EGFR-TKI resistance-related genes in LUAD by bioinformatics methods and aimed to find potential biomarkers for predicting LUAD prognosis. We discovered EGFR-TKI resistance-associated genes using Gene Expression Omnibus (GEO) datasets and developed a five-gene EGFR-TKI resistance-associated gene signature (ERS) using the LUAD dataset from The Cancer Genome Atlas (TCGA). According to ERS-calculated risk scores, TCGA-LUAD patients were accurately classified into two groups: high-risk and low-risk. The ERS was found to be an independent prognostic factor for LUAD patients compared to other clinical pathological markers. Moreover, functional and pathway enrichment studies suggested that the differentially expressed genes between the two risk groups are primarily involved in immunological activities. Further immune cell analyses also suggested that the ERS and immune cells infiltration were closely linked. Interestingly, we found the differential expression of multiple immunotherapy response biomarkers between the two risk groups. We also found that patients with a high risk score had more gene mutation changes and lower IC50 levels for chemotherapy drugs like docetaxel, gemcitabine, sorafenib, and tipifarnib than patients with a low risk score. The verification of the ERS by GSE30219, GSE11969 and GSE72094 also showed a favorable prognostic value for LUAD patients. We considered that the construction of our five-gene signature will help predict the prognosis and potential mechanism studies of LUAD and contribute to optimized therapeutic strategies.

## Materials and methods

### Patients and datasets

The gene expression files of gefitinib-sensitive and gefitinib-resistant lung cancer cell lines were obtained from the GSE60189 and GSE122005 datasets (14, 15). The GSE80344 dataset was used to retrieve the expression profile data of erlotinib-sensitive and erlotinib-resistant lung cancer cell lines (16). All the data above were acquired from the Gene Expression Omnibus (GEO) database (https://www.ncbi.nlm.nih.gov/geo/; May, 15, 2021). The gene expression data and clinical details of LUAD patients were extracted from the TCGA database (https://portal.gdc.cancer.gov/; May, 28, 2021). From the TCGA-LUAD dataset, 535 tumor specimens and 59 surrounding normal tissues were ultimately screened for additional investigation. The gene expression levels and clinical information of GSE30219 (n=83), GSE11969 (n=90) and GSE72094 (n=389) were obtained from GEO database for validating the risk model. All data downloaded from GEO and TCGA were normalized and used for subsequent analyses using R software (17).

### Identification of EGFR-TKI resistance-related genes

A differential expression analysis of TKI-sensitive and TKI-resistant LUAD clones (gefitinib: PC9 and HCC827; erlotinib: HCC827 and HCC4006) was used to identify EGFR-TKI resistance-associated genes (14–16). The differential analyses were conducted using the “limma” R package, with false discovery rate (FDR) <0.05 and absolute log2fold change (|log2FC|) >1. Addition, the “venn” R packages was used to confirm the intersection genes in different cell clones of gefitinib and erlotinib and create the venn diagram. Finally, the aforementioned intersection genes were regarded as EGFR-TKI resistance-related genes for further analyses.

### Construction of the ERS

After downloading the data of LUAD patients from TCGA, EGFR-TKI resistance-related genes were examined using the “limma” R package to identify differentially expressed EGFR-TKI resistance-related genes between the normal and tumor groups. Significant genes were determined to have FDR<0.05 and |log2FC| > 1. Based on the endpoint of patients’ death, TCGA-LUAD cohort was then randomly divided into a training set and a testing set in a ratio of 1:1 by using the receiver-operator characteristic (ROC) curves. We determined the two sets until the two sets reached their best predictive value. Next, univariate and multivariate Cox regression analysis was done on the training cohort to build the five-gene model. P<0.05 was deemed statistically significant. Among the genes associated with ERS, hazard ratio (HR) >1 was considered a risk factor, whereas HR<1 was considered a protective factor. In addition, LUAD patients were separated into low-risk and high-risk groups based on the risk scores calculated by the ERS (the cutoff value was set at 1). Additionally, the “survivalROC” R package was used to generate ROC curves for the three sets of the TCGA-LUAD cohort: the training set, the testing set, and the total set.

### Survival analyses of the high-risk and low-risk groups

The “pheatmap” R package was then used to compare the gene expression, risk score and survival time distribution of ERS in the two risk categories. Additionally, the association between ERS and survival time was evaluated using the “survival” and “survminer” R packages. All aforementioned comparison analyses were performed in the three sets of TCGA-LUAD cohort. Log-rank test were used to compare the difference of overall survival time in the two-risk groups.

### Independent prognostic value evaluation of the ERS

After acquiring the clinicopathological data of LUAD patients, the risk score of ERS and other traditional clinical parameters (age, sex, stage, T stage, N stage) were incorporated in the univariate and multivariate analyses of prognosis. The M stage was not included because that information was unknown for many patients. The “survivalROC” R package was then used to display the predictive value of ERS relative to other clinicopathological variables in the three TCGA-LUAD cohort sets.

### Correlation analyses of clinicopathological factors with the ERS

LUAD patients were separated into distinct subgroups based on their clinical features (age, gender, stage, T stage, and N stage) for the comparison study involving the ERS. This was performed through the combined analysis by the “limma” and “ggpubr” R packages. Additionally, these results were summarized with a heatmap through the R package of “ComplexHeatmap”. The comparison analyses of the clinical factors in different risk groups were assessed by Wilcoxon signed-rank test.

### Functional enrichment analyses

To explore the putative molecular mechanisms linked with the prognostic signature of ERS, Gene Ontology (GO) and Kyoto Encyclopedia of Genes and Genomes (KEGG) pathway enrichment analyses were performed (18). The “limma” R package was used to determine the differential gene expressions between the two risk groups (the cutoffs were FDR<0.05 and |log2FC|>1). A bar plot was drawn using “clusterProfiler,” “org.Hs.eg.db,” “enrichplot,” and “ggplot2” R packages with the following filter conditions: Q<0.05 or P<0.05. The GO analysis mainly evaluated the biological process, cellular component, and molecular function; the results of top 10 were shown. The KEGG analysis mainly evaluated the differentially expressed pathways in different risk groups; the results of top 30 were shown.

### Immune infiltration analyses of different risk groups

Various software, most notably CIBERSORT and single-sample gene set enrichment analysis (ssGSEA), were used to analyze the expression levels of immune cells and immunological functions (19). Using ssGSEA, the infiltration scores of immune cells and immunological functions for LUAD patients were evaluated. Various tools, such as XCELL, TIMER, QUANTISEQ, MCPCOUNTER, EPIC, CIBERSORT-ABS, and CIBERSORT, were utilized to validate the immune cell distinctions of ERS further (20). Additionally, a correlation heatmap of CIBERSORT was used to examine the relationships between immune cells by the Wilcoxon signed-rank test. TCGA-LUAD patients’ gene expressions for immunotherapy response biomarkers PD-1, PD-L1, and CTLA-4 were also analyzed for a thorough study.

### Tumor gene mutation and drug sensitivity analysis of ERS

Then, the “Maftools” R package was used to analyze the tumor mutation burden (TMB) of each sample in the TCGA-LUAD patients. The top 20 genes, which were most frequently mutated in tumor were shown in the waterfall plot. Each column presented a patient. The drug sensitivity of each sample to various drugs was evaluated by using “pRRophetic” R package. The IC50 value of these drugs were compared by the Wilcoxon signed-rank test between groups.

### Single gene expression analyses of ERS

57 patients in the TCGA-LUAD cohort with paired LUAD and normal tissues were screened. The R package was used to find out how the ERS (PCDH7, DEPDC1B, SATB2, S100P, and FGD3) were expressed differently between the LUAD and normal tissues. Next, quantitative real-time PCR (qRT-PCR) was utilized to confirm the gene expression levels of ERS in 24 pairs of surgically resected, frozen LUAD tissues and neighboring normal tissues. RNA was extracted using Trizol reagent (Sigma, USA), and GAPDH served as an internal control for ERS. The relative mRNA expression levels of the five genes were measured using the 2^-ΔΔCt^ technique. The primer sequences utilized in this study were as follows: GAPDH: forward 5′-ACAACTTTGGTATCGTGGAAGG-3′ and reverse 5′-GCCATCACGCCACAGTTTC-3′; PCDH7: forward 5′- TGATCTTCGACGAGAACGAGT-3′ and reverse 5′- CGTTGATGTCAAGCACGATGA -3′; FGD3: forward 5′- AAGATGTACGGCGAGTATGTCA-3′ and reverse 5′- GGAGCCTCTTCAGATAGTCCTT-3′; DEPDC1B: forward 5′- CTGAAGTGACCCGCAAACAAA-3′ and reverse 5′- CTGGTGGGAGATCATTCCATTC-3′; SATB2: forward 5′- GACAGTGGCCGACATGCTAC-3′ and reverse 5′- AGGCAAGTCTTCCAACTTTGAA -3′; S100P: forward 5′- AAGGATGCCGTGGATAAATTGC-3′ and reverse 5′- ACACGATGAACTCACTGAAGTC-3′. The protein expression levels of the ERS in normal lung tissues and LUAD tissues were evaluated through the Human Protein Atlas (HPA) database (https://www.proteinatlas.org/; April 4, 2022).

### Statistical analysis

All bioinformatics data were processed using R (version 3.6.4) and the respective R packages. The experimental data and immunotherapy response biomarkers analysis were performed using GraphPad Prism version 8.0 software (La Jolla, CA, USA). Mann-Whitney test was used for data analysis. P value < 0.05 was considered statistically significant.

## Results

### Construction of the ERS for predicting prognoses

**Figure 1:**
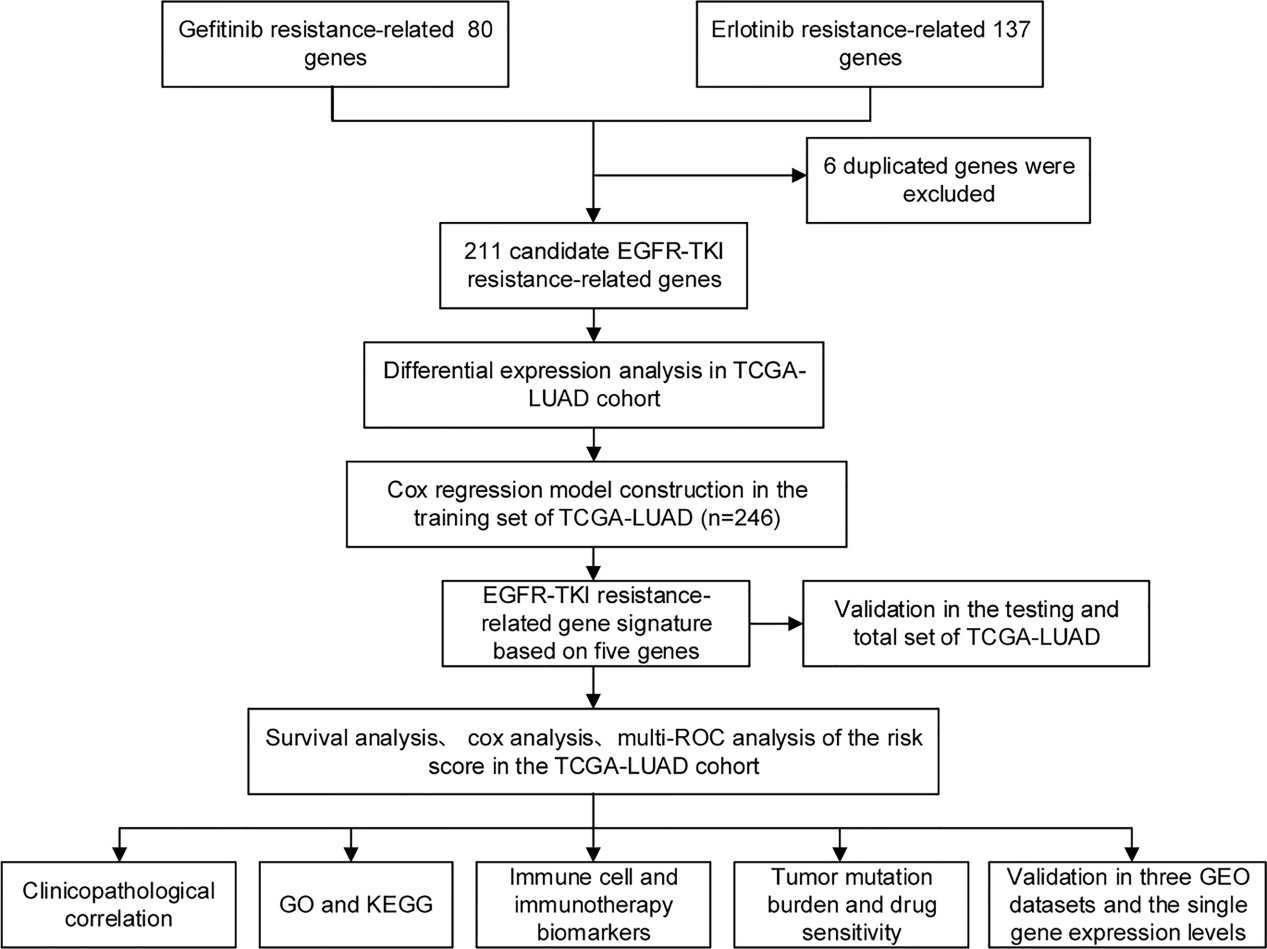
The flow of this study. From GEO databases, genes associated with EGFR-TKI resistance were identified and subsequently studied in the TCGA-LUAD cohort. Cox regression analysis was used to establish the risk model. Based on the computed risk scores of the risk model, TCGA-LUAD patients were divided into high-risk and low-risk groups. The differential analyses among the two groups, such as immune cells and immunotherapy response biomarkers, were explored.

**Figure 2:**
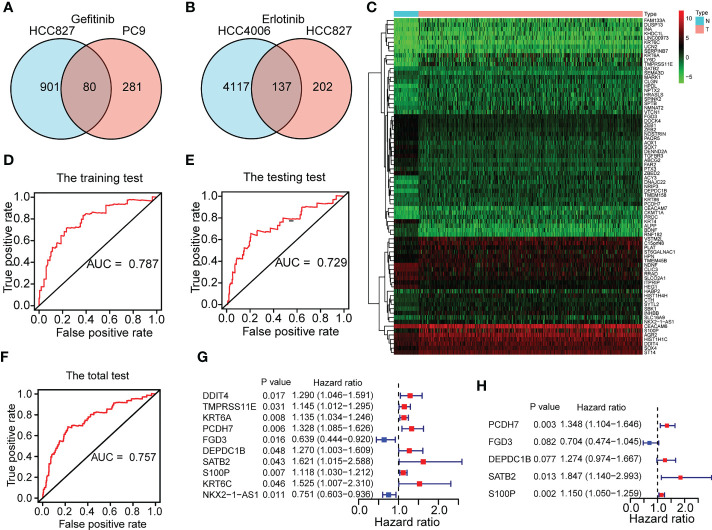
Construction of a five-gene ERS for predicting prognoses. **(A)** 80 gefitinib resistance-related genes were identified through the GEO datasets of GSE60189 and GSE122005. **(B)** 137 erlotinib resistance-related genes were identified through GSE80344. **(C)** 77 differentially expressed EGFR-TKI resistance-related genes were identified between the normal (N) and tumor (T) groups of TCGA-LUAD cohort. **(D–F)** The training set and testing set of TCGA-LUAD cohort respectively for risk model construction and validation were chosen until the ROC curves of the two sets reached a best predictive value. The ROC curve in the total set of TCGA-LUAD cohort was also shown. **(G–H)** Five genes (FGD3, PCDH7, DEPDC1B, SATB2, and S100P) were selected for risk model development based on univariate and multivariate Cox regression analyses conducted on the training set of the TCGA-LUAD cohort.

**Figure 1** depicts the flow pertaining to this investigation. A total of 211 EGFR-TKI resistance-related genes from the GSE60189, GSE122005 and GSE80344 datasets were screened, including 80 gefitinib resistance-related genes and 137 erlotinib resistance-related genes (**Figures 2A, B**). Six genes were excluded because they were identified as both gefitinib and erlotinib resistance-related genes. Finally, 211 genes were selected for further analysis (**Supplementary Table 1**). To learn more about these genes in the TCGA-LUAD cohort, 77 EGFR-TKI resistance-related genes with different levels of expression were found (**Figure 2C** and **Supplementary Table 2**). Of these, 52 were upregulated and 25 were downregulated.

In the TCGA-LUAD cohort, data on the OS of 490 individuals was eventually discovered. Based on the OS time, the TCGA-LUAD cohort (n=490) was randomly divided into the training set (n=246) and testing set (n=244) (**Supplementary Table 3**) with a ratio of 1:1 by running a ROC analysis. Lastly, we determined the training set and testing set until the area under the curve (AUC) values of the ERS in the two sets achieved the highest predictive value. As illustrated in **Figures 2D–F**, the AUC for the training set was 0.787, the AUC for the testing set was 0.729, and the AUC for the entire cohort was 0.757. The baseline features among the three sets (training set, testing set, total set) for LUAD patients were summarized in **Table 1**.

**Table 1:** The baseline characteristics of LUAD patients in the three sets of TCGA cohort.

Clinical characteristics	TCGA-training set (n=246)	TCGA-testing set (n=244)	TCGA in total set (n=490)
Survival status, n (%)			
alive	168 (68.3%)	161 (66.0%)	329 (67.1%)
dead	78 (31.7%)	83 (34.0%)	161 (32.9%)
Age, median (range)			
>65	72 (66~87)	73 (66~88)	72 (66~88)
<=65	58 (38~65)	59 (33~65)	59 (33~65)
Gender, n (%)			
female	132 (53.7%)	134 (54.9%)	266 (54.3%)
male	114 (46.3%)	110 (45.1%)	224 (45.7%)
Stage, n (%)			
stage I	132 (53.6%)	129 (52.9%)	261 (53.3%)
stage II	56 (22.8%)	61 (25.0%)	117 (23.9%)
stage III	40 (16.3%)	39 (16.0%)	79 (16.1%)
stage IV	13 (5.3%)	12 (4.9%)	25 (5.1%)
unknown	5 (2.0%)	3 (1.2%)	8 (1.6%)
T, n (%)			
T1	89 (36.2%)	77 (31.6%)	166 (33.9%)
T2	124 (50.4%)	134 (54.9%)	258 (52.6%)
T3	20 (8.1%)	25 (10.2%)	45 (9.2%)
T4	11 (4.5%)	7 (2.9%)	18 (3.7%)
unknown	2 (0.8%)	1 (0.4%)	3 (0.6%)
N, n (%)			
N0	162 (65.9%)	155 (63.5%)	317 (64.7%)
N1	46 (18.7%)	46 (18.9%)	92 (18.8%)
N2	32 (13.0%)	36 (14.7%)	68 (13.9%)
N3	2 (0.8%)	0	2 (0.4%)
unknown	4 (1.6%)	7 (2.9%)	11 (2.2%)
M, n (%)			
M0	163 (66.3%)	159 (65.2%)	322 (65.7%)
M1	12 (4.9%)	12 (4.9%)	24 (4.9%)
unknown	71 (28.8%)	73 (29.9%)	144 (29.4%)

Based on the clinical objective of OS, the univariate Cox proportional hazards regression analysis revealed 10 differentially expressed EGFR-TKI resistance-related genes to be significant in the training set (**Figure 2G** and **Supplementary Table 4**) (P<0.05). Using a multivariable Cox regression analysis, five genes were finally chosen for model design (**Figure 2H** and **Supplementary Table 5**). Among the five genes identified, an HR >1 was defined as a factor at risk (PCDH7, DEPDC1B, SATB2, and S100P), and an HR <1 was defined as a factor with protective role (FGD3). The following formula was used to calculate the risk score for each sample based on the expression levels of the five genes: risk score = 0.298386164 × PCDH7 + (-0.351049257) × FGD3 + 0.242280512 × DEPDC1B + 0.613634016 × SATB2 + 0.139662184 × S100P. With a median risk score of 1, all patients in the TCGA-LUAD cohort were categorized into distinct risk groups.

### Characteristics and survival analyses of the ERS

**Figure 3:**
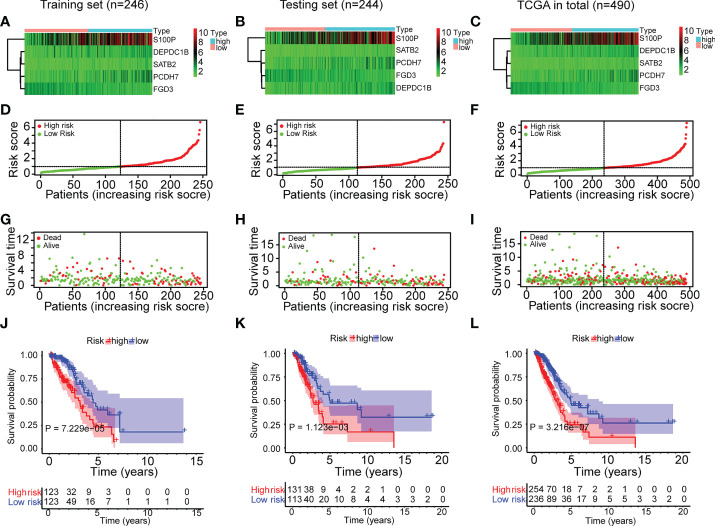
Characteristics and survival analyses of the ERS in the three sets of TCGA-LUAD cohort. **(A–C)** The gene expression heatmap of the ERS in the three sets of TCGA-LUAD cohort. **(D–F)** The risk score distribution plot indicated that the ERS effectively categorized TCGA-LUAD patients into high-risk and low-risk groups. **(G–I)** The survival time distribution plot indicated that the high-risk group had more fatalities and shorter survival times. **(J–L)** The Kaplan-Meier survival study demonstrated the disparity in survival status and survival time between the two risk groups.

To gain a deeper understanding of the strength of the ERS, the features of the ERS and its association with OS in high-risk and low-risk groups were investigated. The distributional comparisons of gene expression levels, risk score, survival time, and survival status between the two groups are depicted in **Figure 3**. As described in **Figures 3A–C**, there were higher levels of PCDH7, DEPDC1B, SATB2, S100P and lower expression of FGD3 in high-risk patients. The ERS effectively categorized TCGA-LUAD patients into high-risk and low-risk groups (**Figures 3D–F**). In addition, the high-risk group had more deceased patients and a shorter survival period (**Figures 3G–I**). Moreover, the Kaplan-Meier analysis revealed a significant association between ERS and poor OS (**Figures 3J–L**) (P <0.01). All of the aforementioned analyses were conducted on the training test, testing set and total set of the TCGA-LUAD cohort and yielded identical results.

### Independent prognostic power evaluation of the ERS

**Figure 4:**
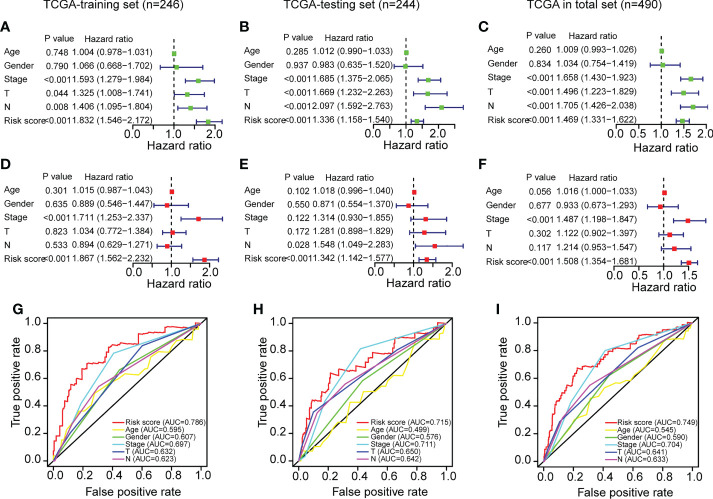
Independent prognostic power evaluation of the ERS in the three sets of TCGA-LUAD cohort. **(A–C)** Analyses of univariate Cox regression revealed that both the risk score and clinical variables were associated with a poor prognosis. **(D–F)** Multivariable Cox regression analyses confirmed the independent prognostic value of the ERS compared to other clinical factors. **(G–I)** Multivariate ROC analysis indicated that the risk score had prognostic predictive significance for LUAD patients.

After determining the ERS’s prognostic value, we assessed its independent prognostic value. As seen in **Figures 4A–C**, univariate Cox regression analysis revealed that ERS and clinical variables (stage, T stage, and N stage) were substantially linked with poor OS in the training set (HR>1; P<0.001). Moreover, a multivariate analysis indicated that the ERS had an independent prognostic value for LUAD patients (**Figures 4D–F**) (P<0.001). Age and gender variations in the OS were not evident (**Figures 4A–F**) (P>0.05). In addition, a multiple ROC curve analysis was done to examine the prognostic prediction abilities of the ERS and clinical variables. As depicted in **Figures 4G–I**, the AUC values of the ERS were greater than those of all the clinical factors in the training set of the TCGA-LUAD cohort, indicating that the ERS had superior prognostic predictive value for LUAD. All of the analyses above were also validated on the testing set andtotal set of the TCGA-LUAD cohort and showed identical results.

### Clinicopathological association of the ERS

**Figure 5:**
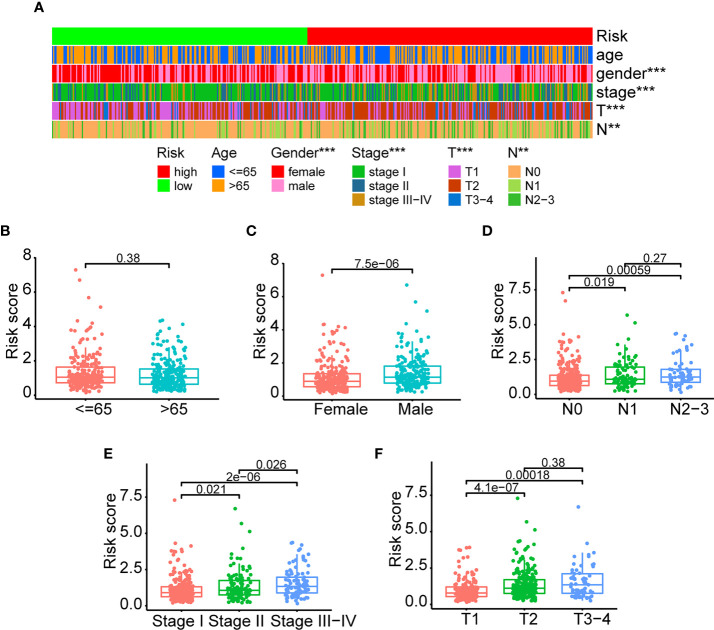
Clinicopathological association of the ERS. **(A)** An overview of graphs depicting the relationship between the ERS and clinical characteristics such as age, gender, stage, T-stage, and N-stage. **(B–F)** The risk score differences were compared among different clinical subgroups (<=65 vs >65; female vs male; stage I vs stage II vs stage III-IV; T1 vs T2 vs T3-4; N0 vs N1 vs N2-3). (**P<0.01, and ***P<0.001).

As previously stated, the ERS and clinical indicators are crucial prognostic variables for LUAD. In addition, we investigated the relationship between ERS and clinicopathological variables. The clinical information and risk score of TCGA-LUAD patients were summarized in **Supplementary Table 6**. There were significant relationships between the ERS and sex, stage, T stage, and N stage, as shown in **Figure 5A**. It is demonstrated that male patients had greater risk scores than female patients (**Figure 5C**) (P<0.05). Additionally, advanced-stage LUAD patients tended to have higher risk scores (**Figures 5D–F**). As shown in **Figure 5D**, patients with stages N1 and N2–3 had higher risk scores than those with stage N0 (P<0.05). Patients with stages II and III–IV had greater risk scores than those with stage I (**Figure 5E**) (P<0.05). Patients with stages T2 and T3–4 had higher risk scores than those with stage T1 (**Figure 5F**) (P<0.01). In conclusion, all of the aforementioned studies suggested that the ERS was associated with a variety of clinicopathological variables. However, the risk score values were comparable between older and younger patients (**Figure 5B**) (P=0.38).

### GO functional enrichment and KEGG pathway enrichment analyses

**Figure 6:**
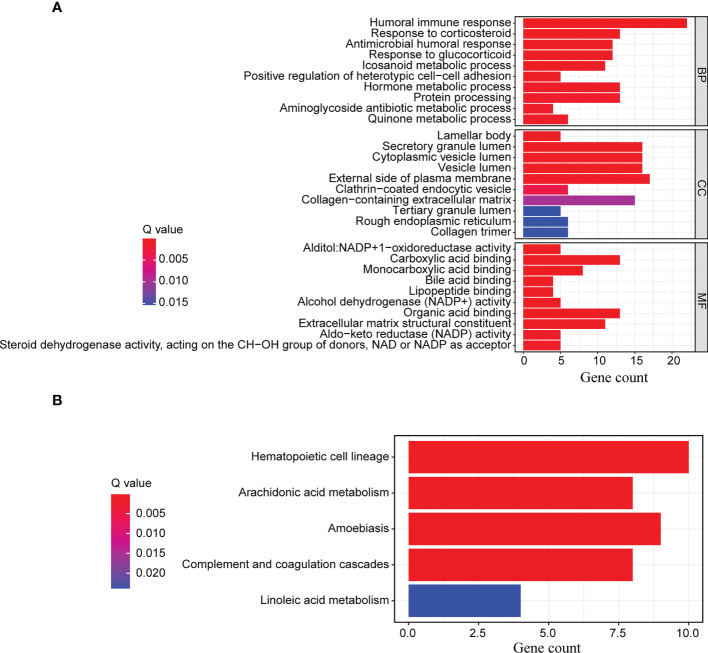
GO functional enrichment and KEGG pathway enrichment analyses. The differentially expressed genes between the high-risk and low-risk groups were further enriched for **(A)** GO functional analysis and **(B)** KEGG pathway analysis.

After identifying the differentially expressed genes between the high-risk and low-risk groups, we ran GO functional enrichment and KEGG pathway enrichment analyses to investigate the putative biological activities and signaling pathways of ERS. According to the results of the GO analysis, the genes were primarily involved in humoral immune response, response to corticosteroids, hormone metabolic process, protein processing, antimicrobial humoral response, and response to glucocorticoids (**Figure 6A** and **Supplementary Table 7**). The KEGG analysis revealed that the majority of the genes were associated with hematopoietic cell lineage, arachidonic acid metabolism, amoebiasis, complement and coagulation cascades, and linoleic acid metabolism (**Figure 6B** and **Supplementary Table 8**). All of these findings suggested that the ERS was primarily associated with hematopoietic cell lineage and the cellular immune response process, and that it may function *via* immune-related pathways.

### Immune infiltration analyses of the high-risk and low-risk groups

**Figure 7:**
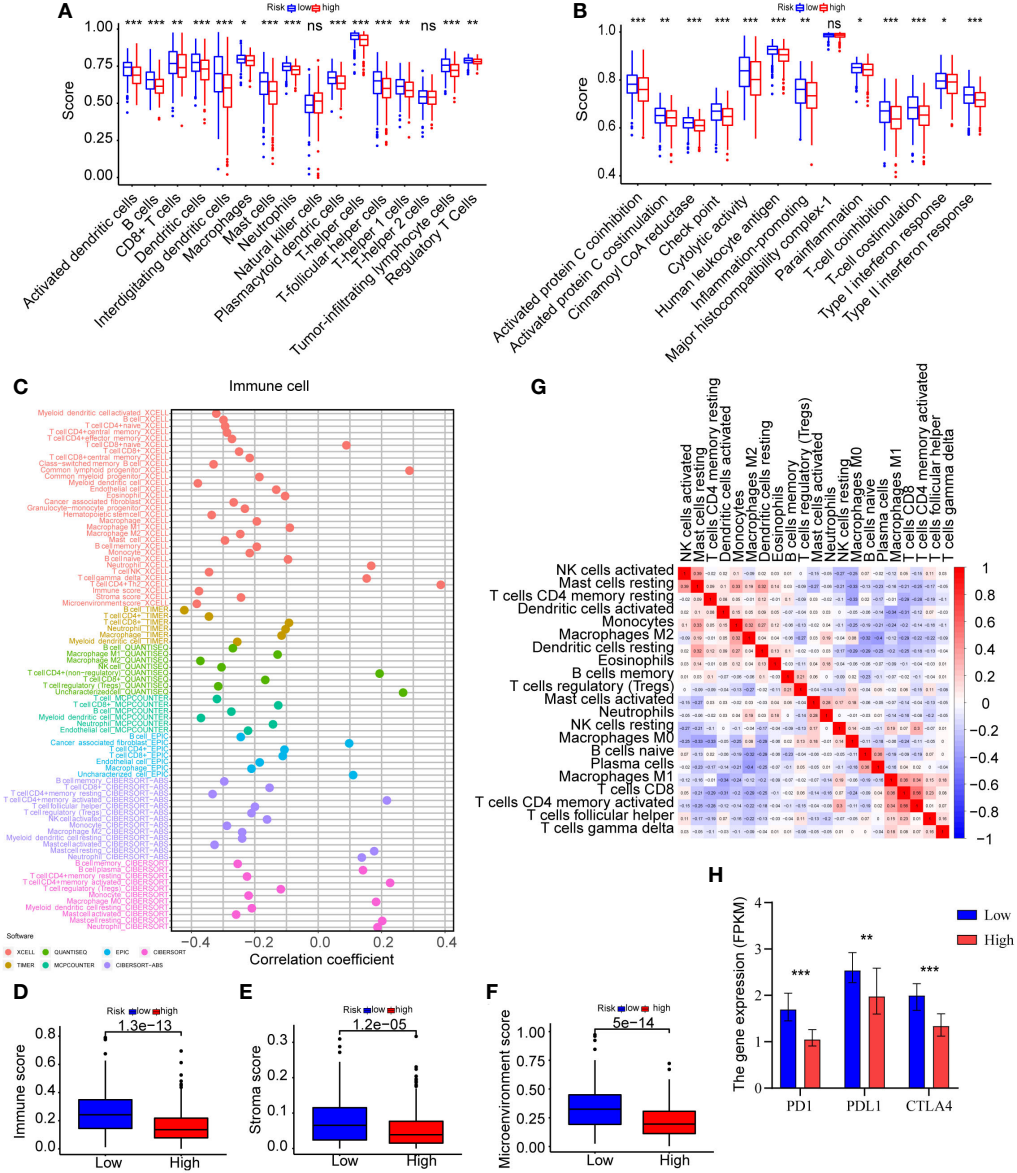
Analyses of immune infiltration in the high-risk and low-risk categories. **(A)** ssGSEA was used to undertake the immune cells score between the two groups. **(B)** The immune cell function analysis among the two groups was explored by ssGSEA. **(C)** Immune cells expression analysis was performed through various methods, including XCELL, TIMER, QUANTISEQ, MCPCOUNTER, EPIC, CIBERSORT-ABS, and CIBERSORT. **(D–F)** The immune score, stroma score and microenvironment score were compared between the two risk groups by XCELL. **(G)** CIBERSORT was used for the association analysis between immune cells. **(H)** The expression levels of PD-1, PD-L1, and CTLA-4 were compared between the two groups (*P<0.05, **P<0.01, and ***P<0.001).

To further examine the immune status of high-risk and low-risk groups, we assessed the expression of immune cells in LUAD patients using ssGSEA algorithms and a variety of tools, such as XCELL, TIMER, QUANTISEQ, MCPCOUNTER, EPIC, and CIBERSORT-ABS. The ssGSEA data indicated that the difference in immune infiltration between the two risk groups was statistically significant (**Figures 7A, B** and **Supplementary Table 9**). The infiltration scores of diverse immune cells, including activated dendritic cells, B cells, dendritic cells, interdigitating dendritic cells, mast cells, neutrophils, plasmacytoid dendric cells, T-helper cells, T-follicular helper cells, and tumor-infiltrating lymphocyte cells, were significantly lower in the high-risk group compared to the low-risk group (**Figure 7A**) (P<0.001). Moreover, we found comparable differences in infiltration scores based on immune cell functions between low-risk and high-risk patients, including those of activated protein C coinhibition, cinnamoyl CoA reductase, check point, cytolytic activity, human leukocyte antigen, T-cell coinhibition, T-cell costimulation, and type II interferon response (**Figure 7B**) (P<0.001). The lower score for immune cell infiltration indicates that high-risk patients may have an immunosuppressive microenvironment. We further studied the relationship between the differential expression of immune cells and the risk score using a variety of techniques for analyzing immune cells. In accordance with the findings of the ssGSEA, various immune cells exhibited a negative connection with the risk score (**Figure 7C** and **Supplementary Table 10**). Evidently, the immune score, stroma score and microenvironment score are lower in high-risk groups (**Figures 7D–F**). Moreover, correlation analysis between various immune cells using CIBERSORT revealed that activated natural killer cells, resting mast cells, resting memory CD4 T-cells, activated dendritic cells, monocytes, M2 macrophages, resting dendritic cells were negatively correlated with resting natural killer cells, M0 macrophages, naïve B cells, plasma cells, M1 macrophages, CD8 T-cells, activated memory CD4 T-cells and T-follicular helper cells (**Figure 7G** and **Supplementary Table 11**). In summary, these findings indicated a substantial relationship between the ERS and the immune microenvironment of tumors.

Considering the immunological relationship of the ERS, we examined the immunotherapy response biomarker expressions further. It is recognized that PD-1, PD-L1, and CTLA-4 are useful biomarkers for predicting the response to immunotherapy (21), and their high expressions may indicate the need for future immunotherapy applications. As displayed in **Figure 7H** and **Supplementary Table 12**, the findings of all gene expression studies revealed lower expressions of PD-1 (P<0.001), PD-L1 (P=0.005), CTLA-4 (P<0.001) in high-risk patients, suggesting a possible link between the ERS and a poor immunotherapy response. The analysis results were summarized in **Table 2**. However, this could not be proven due to insufficient data.

**Table 2:** The differential expression analysis results of PD1, PDL1, and CTLA4 in the high-risk and low-risk patients of TCGA-LUAD.

Gene	Mean		Median		Difference between medians		95%CI	*P value
	Low-risk	High-risk	Low-risk	High-risk	Difference: Actual	Difference: Hodges-Lehmann	
PD1	2.426	1.775	1.695	1.048	-0.647	-0.453	-0.679 **–** -0.239	<0.001
PDL1	4.145	4.238	2.533	1.976	-0.557	-0.469	-0.78 **–** -0.142	0.0054
CTLA4	2.541	1.832	1.995	1.337	-0.658	-0.562	-0.808 **–** -0.321	<0.001

*Mann-Whitney test was used for data analysis.

### Tumor gene mutation and drug sensitivity of the ERS

**Figure 8:**
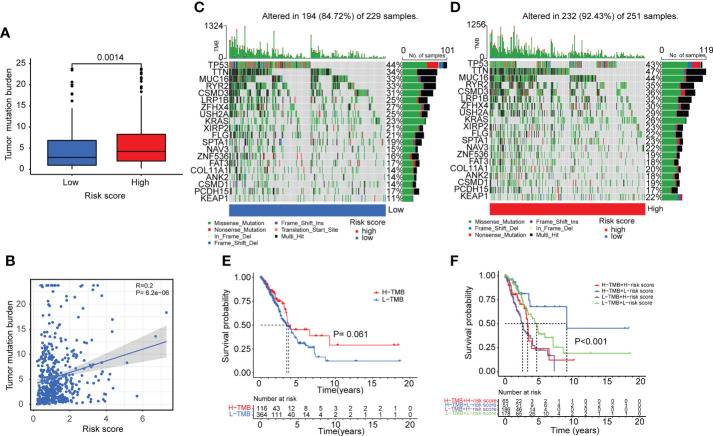
Tumor mutation burden analyses in lung adenocarcinoma patients. **(A, B)** Boxplot and correlation plot showed the relation of tumor somatic mutation with risk score in lung adenocarcinoma patients. **(C, D)** The summary charts illustrated the distribution of the top 20 tumor somatic mutations in the high-risk and low-risk groups. **(E)** The survival status and survival time difference between the high-TMB and low-TMB groups. **(F)** The Kaplan-Meier analysis showed the survival status and survival time in four subgroups: H-TMB+H-risk score, H-TMB+L-risk score, L-TMB+H-risk score, L-TMB+L-risk score.

The TMB of the high-risk and low-risk groups was then compared. As illustrated in **Figures 8A, B**, patients in the high-risk group had greater TMB levels. A review of gene mutation modification frequencies suggested that the high-risk group had a gene mutation rate of 92.43%, while the low-risk group was 84.72% (**Figures 8C, D**). In the high-risk group, the top five genes were TTN (47%), MUC16 (44%), TP53 (43%), CSMD3 (36%) and RYR2 (35%). In general, the mutation rate of oncogenes, such as TTN, was greater in high-risk individuals (47% vs. 34%), although the mutation rate of anti-oncogenes, such as TP53, was comparable in both groups (43% vs.44%). Then, based on the optimal cutoff of TMB by the R packages, the patients were stratified into high-TMB and low-TMB groups. However, there was no significant difference in survival between the two groups (**Figure 8E**) (P>0.05). Moreover, compared to the other three groups (H-TMB+H-risk score, H-TMB+L-risk score, and L-TMB+L-risk score), patients with low TMB and high risk score (L-TMB+H-risk score) had significantly shorter survival times (**Figure 8F**).

**Figure 9:**
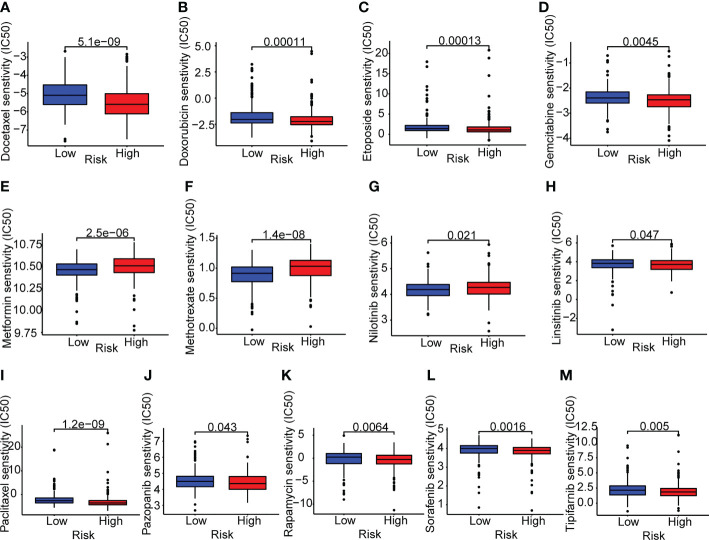
Drug sensitivity analyses in lung adenocarcinoma patients. **(A–M)** Boxplots of the two risk groups showed the mean IC50 differences of 13 representative drugs (docetaxel, doxorubicin, etoposide, gemcitabine, metformin, methotrexate, nilotinib, linsitinib, paclitaxel, pazopanib, rapamycin, sorafenib, tipifarnib).

Next, we investigated the variations in medication sensitivity between the two groups. The IC50 values of 138 chemotherapeutic agents and inhibitors were determined and compared between the two risk categories. As shown in **Figures 9A–M**, thirteen sample medications were selected. LUAD patients at high risk had lower IC50 values of docetaxel, doxorubicin, etoposide, gemcitabine, linsitinib, paclitaxel, pazopanib, rapamycin, sorafenib, and tipifarnib than patients with a low risk, which suggested that those drugs may be acceptable therapy for high-risk patients, according to the data. In contrast, patients at high risk had higher IC50 values of metformin, methotrexate, and nilotinib, which implied that those medications may not be suitable for high-risk patients.

### External validation and the gene expression analysis of the ERS

**Figure 10:**
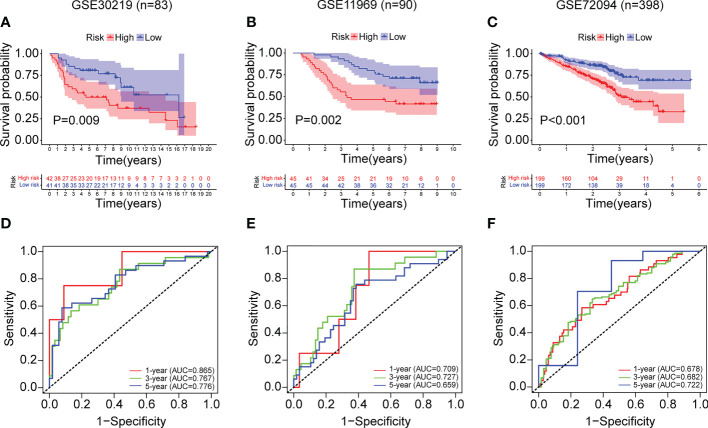
External validation of the ERS in the GSE30219, GSE11969 and GSE72094 datasets. **(A–C)** The Kaplan-Meier survival analysis of LUAD patients in the two risk groups divided by the ERS in the datasets of GSE30219 **(A)**, GSE11969 **(B)**, and GSE72094 **(C)**. **(D–F)** The ROC curves of the ERS for predicting the 1-, 3- and 5-year survival in the datasets of GSE30219 **(D)**, GSE11969 **(E)**, and GSE72094 **(F)**.

Based on the median risk score, we further validated the risk model by GSE30219 (n=83), GSE11969 (n=90) and GSE72094 (n=398). As shown in **Figures 10A–C**, the Kaplan-Meier analysis showed that patients in the high-risk group had poorer survival time compared to the low-risk group in GSE30219 (P=0.009), GSE11969 (P=0.002), GSE72094 (P<0.001). In addition, the ROC analysis showed an acceptable prognostic value for LUAD patients (GSE30219: 1-year AUC = 0.865, 3-year AUC = 0.767, 5-year AUC = 0.776; GSE11969: 1-year AUC = 0.709, 3-year AUC = 0.727, 5-year AUC = 0.659; GSE72094: 1-year AUC = 0.678, 3-year AUC = 0.682, 5-year AUC = 0.722) (**Figures 10D–F**).

**Figure 11:**
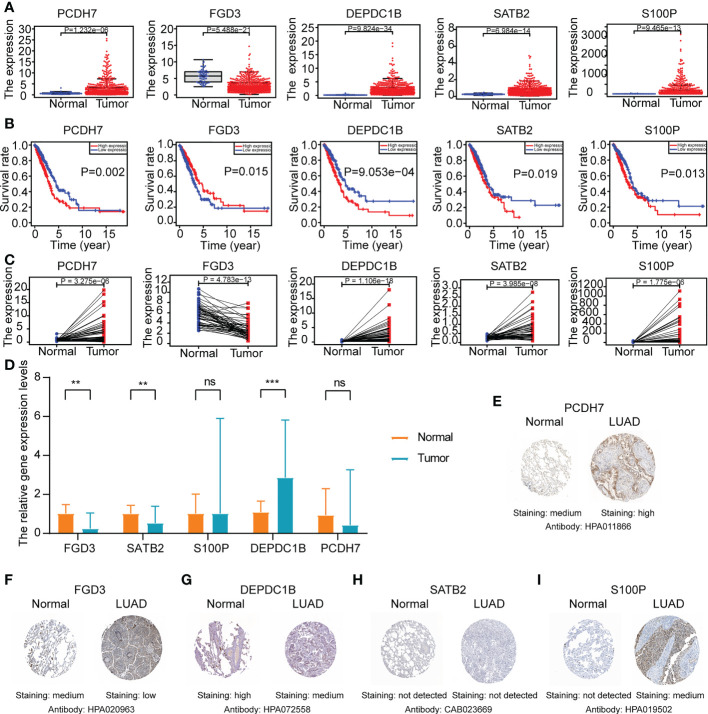
The gene expression levels of ERS (FGD3, PCDH7, DEPDC1B, SATB2, and S100P) in normal and LUAD tissues. **(A)** The gene expression level analyses of ERS in normal and LUAD tissues of TCGA-LUAD cohort. **(B)** The survival rate analyses of ERS in TCGA-LUAD patients. **(C)** The gene expression level analyses of ERS in paired lung cancer tissue and adjacent normal tissue of TCGA-LUAD cohort. **(D)** Relative mRNA expression levels of ERS in normal and LUAD tissues through qRT-PCR analysis (*P<0.05, **P<0.01, and ***P<0.001). **(E–I)** Representative Immunohistochemistry images of ERS in normal lung tissue and LUAD tissue from the HPA database.

Then, the gene expression levels of the ERS (PCDH7, DEPDC1B, SATB2, S100P, and FGD3) were confirmed using various techniques. As demonstrated in **Figure 11A**, LUAD tissues from the TCGA cohort had a greater expression level of PCDH7, DEPDC1B, SATB2, and S100P and a lower expression level of FGD3. Evidently, based on the median of the gene expression levels, we found that the levels for five genes were related with LUAD prognoses (**Figure 11B**). Analysis of gene expression in paired lung cancer and surrounding normal tissue from TCGA-LUAD patients indicated a consistent outcome (**Figure 11C**). In addition, the results of qRT-PCR indicated that DEPDC1B was up in LUAD tissues (P<0.001) whereas FGD3 was elevated in normal lung tissues (P<0.01) (**Figure 10D**), which was consistent with the bioinformatics results. Nevertheless, SATB2 levels were lower in LUAD tissues (P<0.01) (**Figure 11D**). PCDH7 and S100P were not statistically significant between the two groups (P>0.05), which may be explained by the small sample size and high individual variation. The protein expression of five genes was evaluated further using the HPA database (**Figures 11E–I**). The results indicated that the expression of PCDH7 and S100P was up in LUAD tissues, while the expression of FGD3 and DEPDC1B was lowered. Moreover, SATB2 was not discovered.

## Discussion

cResistance to EGFR-TKIs remains a prominent problem that limits their clinical application for lung cancer patients with EGFR mutation. With the advancements in bioinformatics technology, a growing number of prognostic biomarkers and therapeutic targets have been identified for LUAD patients (22, 23). However, indicators associated with EGFR-TKI resistance in LUAD patients are scarce. To investigate the potential clinical relevance and molecular processes of EGFR-TKI resistance-related genes in LUAD patients, we developed a risk model of ERS based on 211 EGFR-TKI resistance-related genes identified from three GEO datasets. The TCGA-LUAD cohort was separated into training and testing sets. We created the ERS for the training set of TCGA-LUAD cohort using univariate and multivariate Cox analysis. Moreover, the ERS was well-validated for both the testing set and the full TCGA-LUAD cohort. The multivariate Cox and multi-ROC analyses further confirmed the independent prognostic and predictive usefulness of ERS for LUAD patients in comparison to other routine clinical variables. Interestingly, the GO functional enrichment and KEGG pathway enrichment studies revealed a substantial association between the risk score and immunological processes. Therefore, an examination of immune infiltration was performed on the high-risk and low-risk groups. Various immunological profile approaches revealed an evident relationship between ERS and tumor-infiltrating immune cells, indicating a link between ERS and immune infiltration. In addition, individuals in the high-risk group showed decreased expression levels of numerous indicators of immunotherapy response, indicating a poor immunotherapy response in these patients. Moreover, the high-risk group demonstrated greater TMB and chemotherapeutic medication sensitivity. The ERS was also verified of the by GSE30219, GSE11969 and GSE72094, and showed a favorable prognostic value for LUAD patients. This was the first ERS associated with a poor prognosis in LUAD patients and with a high predictive capacity for treatment responses. Further validation of the putative pathways of the prognostic model is required.

During our investigation, we collected 80 gefitinib resistance-related genes and 137 erlotinib resistance-related genes to gain a comprehensive understanding of EGFR-TKI resistance-related genes in LUAD patients (14–16). Five prognostic biomarkers (FGD3, PCDH7, DEPDC1B, SATB2, and S100P) were identified based on the findings of univariate and multivariate Cox proportional hazards regression analysis. The FGD3 gene is a guanine nucleotide exchange factor that may activate cell division control protein 42 and regulate cell morphology *via* the formation of lamellipodia (24). FGD3 mainly exists in breast cancer and is a promising biomarker of better prognoses for breast cancer patients (24). Dong et al. also identified the potential role of FGD3 in the lncRNA-miRNA-ceRNA network (25); however, its expression and function in lung cancer need to be validated further. PCDH7, also known as cadherin-related neuronal receptor, is a protocadherin family member that primarily acts through homophilic cell-cell contact (26). One study suggested that the expression of PCDH7 was related with lower metastasis-free survival in NSCLC patients and enhanced brain metastasis by facilitating the formation of carcinoma-astrocyte gap junctions (26). Moreover, PCDH7 can synergize with EGFR and KRAS, thus inducing MAPK signaling and lung tumorigenesis (27) and functioning as a potential therapeutic target. DEPDC1B is a cell cycle-controlled protein that builds up during the G2 phase and coordinates cell-cycle progression and de-adhesion during mitosis (28). It is assumed that DEPDC1B is overexpressed in a variety of malignancies and predicts worse patient outcomes (29–31). Upregulation of DEPDC1B is inversely linked with patient survival in NSCLC and may increase tumor cell motility and invasion by activating Wnt/b-catenin signaling (31) and functioning as a potential biomarker. SATB2 is a DNA-binding protein that has an important role in transcriptional regulation and chromatin recombinant (32). SATB2 has been identified as a tumor suppressor and promoter in cancer (33). For NSCLC patients, a low expression SATB2 is related to poor prognoses (32) and may promote tumor progression through epithelial-to-mesenchymal transition (32–34). S100P is a calcium-binding protein that participates in multiple biological processes, including cell cycle progression and differentiation (35). The overexpression of S100P is linked to treatment resistance, metastasis, and negative clinical outcomes (36). However, S100P induction may be considered an important step during the early stage of LUAD; furthermore, its low expression during advanced stages seems to be associated with tumor progression (37). Recent studies have suggested that S100P has an immune role in NSCLC (38, 39). During our analysis, increased expressions of PCDH7, DEPDC1B, SATB2, and S100P were detected in lung cancer tissues and adjacent normal tissue of TCGA-LUAD patients, but expression of FGD3 was observed to be lower. Further validation of qRT-PCR *in vitro* experiment showed that DEPDC1B was higher and FGD3 was lower in LUAD tissues. However, SATB2 was found lower in LUAD tissues, and there was no statistically significance of PCDH7 and S100P between the two groups, which may be explained by the small sample volume and large individual differences. A relatively limited number of studies have focused on these genes and LUAD; therefore, more studies are necessary. The five-gene signature plays a crucial role in LUAD prognosis prediction and may be useful in identifying potential mechanisms.

To provide an understanding of the potential molecular mechanisms and identify new therapeutic targets, we found that the signaling pathways were primarily focused on hematopoietic cell lineage, arachidonic acid metabolism, amoebiasis, complement and coagulation cascades, and linoleic acid metabolism. Hence, a comprehensive immune infiltration analysis was conducted. The infiltration scores of most tumor-infiltrating immune cells were lower for high-risk patients. Considering the relationship between the ERS and the immune processes, we analyzed the expression of immunotherapy response indicators further. All immunotherapy response biomarkers exhibited reduced expression in the high-risk group, which may indicate a poor immunotherapy response for ERS. Moreover, the high-risk patients exhibited a higher tumor mutation burden and treatment sensitivity to a variety of chemotherapeutic agents, such as docetaxel, doxorubicin, etoposide, gemcitabine, linsitinib, paclitaxel, pazopanib, rapamycin, sorafenib, and tipifarnib.

Based on these results, the ERS could independently predict the prognosis for LUAD patients and is associated with immune infiltration. However, this study had some shortcomings. First, there are limited data regarding EGFR-TKIs in the GEO datasets; therefore, it is difficult to screen EGFR-TKI resistance-related genes. More studies of resistance to various EGFR-TKIs are needed. Second, we only preliminarily explored the possible molecular mechanisms of the risk score and its association with immune infiltration. More research is required to validate particular mechanisms.

## Conclusions

This was the first study to demonstrate the expression profiles and potential clinical relevance of EGFR-TKI resistance-related genes in LUAD patients. We also constructed and validated a risk model for predicting poor outcomes and explored associations with immune cells in LUAD patients. These findings may improve disease management in clinical practice and enable optimized immunotherapy for LUAD patients.

## Data availability statement

The original contributions presented in the study are included in the article/**Supplementary Material**. Further inquiries can be directed to the corresponding authors.

## Ethics statement

The collection of tissue samples was approved by the ethical committee of Tongji Medical College, Huazhong University of Science and Technology ([2010]IEC(S202)) and all patients provided informed consent. The patients/participants provided their written informed consent to participate in this study.

## Author contributions

EZ and YJ designed and wrote the manuscript. FW and MG edited the manuscript. ZY and YL handled the partial data. ML, HX, and JD helped create the figures and tables. GY contributed to collect tissue specimens. EZ, FW, MG, ZY, and YL equally contributed to this manuscript. All authors accept responsibility for the work’s contents. All authors contributed to the article and approved the submitted version.

## Funding

This article was made possible through financing from the China National Natural Science Foundation (no. 82070099 and no.81973989). The funding organization had no effect on the content of this article.

## Acknowledgments

We appreciate all the contributions made by the GEO and TCGA databases.

## Conflict of interest

The authors declare that the research was conducted in the absence of any commercial or financial relationships that could be construed as a potential conflict of interest.

## Publisher’s note

All claims expressed in this article are solely those of the authors and do not necessarily represent those of their affiliated organizations, or those of the publisher, the editors and the reviewers. Any product that may be evaluated in this article, or claim that may be made by its manufacturer, is not guaranteed or endorsed by the publisher.

## Abbreviations

EGFR, epidermal growth factor receptor; TKI, tyrosine kinase inhibitor; ERS, EGFR-TKI resistance-related gene signature; LUAD, lung adenocarcinoma; GEO, Gene Expression Omnibus; TCGA, The Cancer Genome Atlas; NSCLC, nonsmall-cell lung cancer; ROC, receiver-operator characteristic; AUC, area under the curve; GO, Gene Ontology; KEGG, Kyoto Encyclopedia of Genes and Genomes; ssGSEA, single-sample gene set enrichment analysis; OS, overall survival; HR, hazard ratio; TMB tumor mutation burden; FDR, false discovery rate.
