# Supplementary material for: Identification of a novel gene signature of lung adenocarcinoma based on epidermal growth factor receptor-tyrosine kinase inhibitor resistance
Source: Front Oncol. 2022 Dec 1;12:1008283. doi: 10.3389/fonc.2022.1008283 (PMC9751970; doi:10.3389/fonc.2022.1008283)
Supplement: Supplementary file 1 [file DataSheet_1.zip › Supplementary_Material/Supplementary_Material.docx]

Supplementary Material

**Supplementary Table 1.** A total of 211 EGFR-TKIs resistance-related genes were identified by the differential expression analysis (submitted as a separate excel file).

**Supplementary Table 2.** Identified 77 differentially expressed EGFR-TKIs resistance-related genes in TCGA-LUAD cohort (related to Figure 2C).

**Supplementary Table 3.** The training (n=246) and testing set (n=244) information of the TCGA-LUAD cohort (submitted as a separate excel file).

**Supplementary Table 4.** The results by univariate cox regression analysis in the training set of TCGA-LUAD cohort (related to Figure 2G).

| **Gene** | **HR** | **HR.95L** | **HR.95H** | **P value** |
| --- | --- | --- | --- | --- |
| DDIT4 | 1.290024 | 1.046121 | 1.590794 | 0.017236 |
| TMPRSS11E | 1.144844 | 1.012205 | 1.294864 | 0.031315 |
| KRT6A | 1.134924 | 1.033662 | 1.246107 | 0.007947 |
| PCDH7 | 1.328178 | 1.084609 | 1.626444 | 0.006038 |
| FGD3 | 0.638818 | 0.443719 | 0.919703 | 0.015946 |
| DEPDC1B | 1.269988 | 1.002596 | 1.608695 | 0.04754 |
| SATB2 | 1.62103 | 1.015328 | 2.588068 | 0.043002 |
| S100P | 1.117657 | 1.030344 | 1.212368 | 0.007357 |
| KRT6C | 1.525191 | 1.007067 | 2.309883 | 0.046237 |
| NKX2-1-AS1 | 0.751243 | 0.603071 | 0.93582 | 0.010719 |

**Supplementary Table 5.** The results of multivariate cox regression analysis in the training set of TCGA-LUAD cohort (related to Figure 2H).

| **Gene** | **Coefficient** | **HR** | **HR.95L** | **HR.95H** | **P value** |
| --- | --- | --- | --- | --- | --- |
| PCDH7 | 0.298386 | 1.347682 | 1.103602 | 1.645744 | 0.003423 |
| FGD3 | -0.351049 | 0.703949 | 0.474221 | 1.044964 | 0.081553 |
| DEPDC1B | 0.242281 | 1.274152 | 0.973952 | 1.666882 | 0.077157 |
| SATB2 | 0.613634 | 1.847132 | 1.14013 | 2.99255 | 0.012678 |
| S100P | 0.139662 | 1.149885 | 1.05037 | 1.258829 | 0.002494 |

**Supplementary Table 6.** The clinical information and risk score of TCGA-LUAD patients (submitted as a separate excel file).

**Supplementary Table 7.** The GO functional enrichment analyses of differentially expressed genes between the high- and low-risk groups in TCGA-LUAD cohort (submitted as a separate excel file).

**Supplementary Table 8.** The KEGG analyses of differentially expressed genes between the high- and low-risk groups in TCGA-LUAD cohort (related to Figure 6B).

| Description | P value | P.adjust | q value | Gene ID | Count |
| --- | --- | --- | --- | --- | --- |
| Hematopoietic cell lineage | 0.0000 | 0.0002 | 0.0002 | HLA-DQB1/HLA-DRB5/IL1R2/CD19/MS4A1  /CD1A/CD1E/CD22/CR2/CD1C | 10 |
| Arachidonic acid metabolism | 0.0000 | 0.0002 | 0.0002 | PTGDS/AKR1C3/PLA2G1B/PLA2G12B/GPX2/  PLA2G4F/CYP4F3/ALOX15 | 8 |
| Amoebiasis | 0.0000 | 0.0009 | 0.0008 | IL1R2/SERPINB4/CD1A/CD1E/SERPINB3/  CTSG/CD1C/LAMA3/LAMC2 | 9 |
| Complement and coagulation cascades | 0.0000 | 0.0013 | 0.0012 | FGG/FGA/C7/CPB2/C4BPA/CR2/SERPIND1/FGB | 8 |
| Linoleic acid metabolism | 0.0008 | 0.0272 | 0.0238 | PLA2G1B/PLA2G12B/PLA2G4F/ALOX15 | 4 |

**Supplementary Table 9.** The immune cell expression analysis through ssGSEA methods in TCGA-LUAD patients (submitted as a separate excel file).

**Supplementary Table 10.** The correlation profiles of the immune cell expression with the risk score through various methods, including TIMER、CIBERSORT、CIBERSORT-ABS、QUANTISEQ、MCPCOUNTER、XCELL、EPIC (related to Figure 7C).

| **Immune cell** | **Correlation** | **P value** | **Immune cell** | **Correlation** | **P value** |
| --- | --- | --- | --- | --- | --- |
| B cell_TIMER | -0.4224 | 0.0000 | Myeloid dendritic cell_MCPCOUNTER | -0.3699 | 0.0000 |
| T cell CD4+_TIMER | -0.3447 | 0.0000 | Neutrophil_MCPCOUNTER | -0.1426 | 0.0016 |
| T cell CD8+_TIMER | -0.0921 | 0.0417 | Endothelial cell_MCPCOUNTER | -0.2214 | 0.0000 |
| Neutrophil_TIMER | -0.1029 | 0.0229 | Myeloid dendritic cell activated_XCELL | -0.3215 | 0.0000 |
| Macrophage_TIMER | -0.1156 | 0.0105 | B cell_XCELL | -0.2988 | 0.0000 |
| Myeloid dendritic cell_TIMER | -0.2553 | 0.0000 | T cell CD4+ naive_XCELL | -0.2935 | 0.0000 |
| B cell memory_CIBERSORT | -0.2539 | 0.0000 | T cell CD4+ central memory_XCELL | -0.2877 | 0.0000 |
| B cell plasma_CIBERSORT | 0.1412 | 0.0017 | T cell CD4+ effector memory_XCELL | -0.2720 | 0.0000 |
| T cell CD4+ memory resting_CIBERSORT | -0.2245 | 0.0000 | T cell CD8+ naive_XCELL | 0.0890 | 0.0493 |
| T cell CD4+ memory activated_CIBERSORT | 0.2269 | 0.0000 | T cell CD8+_XCELL | -0.2496 | 0.0000 |
| T cell regulatory (Tregs)_CIBERSORT | -0.1180 | 0.0090 | T cell CD8+ central memory_XCELL | -0.2161 | 0.0000 |
| Monocyte_CIBERSORT | -0.2197 | 0.0000 | Class-switched memory B cell_XCELL | -0.3301 | 0.0000 |
| Macrophage M0_CIBERSORT | 0.1825 | 0.0000 | Common lymphoid progenitor_XCELL | 0.2880 | 0.0000 |
| Myeloid dendritic cell resting_CIBERSORT | -0.2094 | 0.0000 | Common myeloid progenitor_XCELL | -0.1855 | 0.0000 |
| Mast cell activated_CIBERSORT | -0.2589 | 0.0000 | Myeloid dendritic cell_XCELL | -0.3796 | 0.0000 |
| Mast cell resting_CIBERSORT | 0.2021 | 0.0000 | Endothelial cell_XCELL | -0.1324 | 0.0034 |
| Neutrophil_CIBERSORT | 0.1872 | 0.0000 | Eosinophil_XCELL | -0.1043 | 0.0210 |
| B cell memory_CIBERSORT-ABS | -0.2964 | 0.0000 | Cancer associated fibroblast_XCELL | -0.2667 | 0.0000 |
| T cell CD8+_CIBERSORT-ABS | -0.1534 | 0.0007 | Granulocyte-monocyte progenitor_XCELL | -0.2313 | 0.0000 |
| T cell CD4+ memory resting_CIBERSORT-ABS | -0.3332 | 0.0000 | Hematopoietic stem cell_XCELL | -0.3361 | 0.0000 |
| T cell CD4+ memory activated_CIBERSORT-ABS | 0.2167 | 0.0000 | Macrophage_XCELL | -0.1936 | 0.0000 |
| T cell follicular helper_CIBERSORT-ABS | -0.1996 | 0.0000 | Macrophage M1_XCELL | -0.0895 | 0.0480 |
| T cell regulatory (Tregs)_CIBERSORT-ABS | -0.2105 | 0.0000 | Macrophage M2_XCELL | -0.2458 | 0.0000 |
| NK cell activated_CIBERSORT-ABS | -0.1613 | 0.0003 | Mast cell_XCELL | -0.2941 | 0.0000 |
| Monocyte_CIBERSORT-ABS | -0.2880 | 0.0000 | B cell memory_XCELL | -0.1937 | 0.0000 |
| Macrophage M2_CIBERSORT-ABS | -0.2399 | 0.0000 | Monocyte_XCELL | -0.2159 | 0.0000 |
| Myeloid dendritic cell resting_CIBERSORT-ABS | -0.2399 | 0.0000 | B cell naive_XCELL | -0.0952 | 0.0354 |
| Mast cell activated_CIBERSORT-ABS | -0.3270 | 0.0000 | Neutrophil_XCELL | 0.1672 | 0.0002 |
| Mast cell resting_CIBERSORT-ABS | 0.1766 | 0.0001 | T cell NK_XCELL | -0.3443 | 0.0000 |
| Neutrophil_CIBERSORT-ABS | 0.1376 | 0.0023 | T cell gamma delta_XCELL | 0.1529 | 0.0007 |
| B cell_QUANTISEQ | -0.2692 | 0.0000 | T cell CD4+ Th2_XCELL | 0.3867 | 0.0000 |
| Macrophage M1_QUANTISEQ | -0.1281 | 0.0046 | immune score_XCELL | -0.3762 | 0.0000 |
| Macrophage M2_QUANTISEQ | -0.3716 | 0.0000 | stroma score_XCELL | -0.2436 | 0.0000 |
| NK cell_QUANTISEQ | -0.3050 | 0.0000 | microenvironment score_XCELL | -0.3830 | 0.0000 |
| T cell CD4+ (non-regulatory)_QUANTISEQ | 0.1938 | 0.0000 | B cell_EPIC | -0.2443 | 0.0000 |
| T cell CD8+_QUANTISEQ | -0.1669 | 0.0002 | Cancer associated fibroblast_EPIC | 0.0978 | 0.0307 |
| T cell regulatory (Tregs)_QUANTISEQ | -0.3154 | 0.0000 | T cell CD4+_EPIC | -0.1065 | 0.0185 |
| uncharacterized cell_QUANTISEQ | 0.2679 | 0.0000 | T cell CD8+_EPIC | -0.1118 | 0.0134 |
| T cell_MCPCOUNTER | -0.3195 | 0.0000 | Endothelial cell_EPIC | -0.1846 | 0.0000 |
| T cell CD8+_MCPCOUNTER | -0.1261 | 0.0053 | Macrophage_EPIC | -0.2104 | 0.0000 |
| B cell_MCPCOUNTER | -0.2739 | 0.0000 | uncharacterized cell_EPIC | 0.1103 | 0.0147 |

**Supplementary Table 11.** The correlation analysis between immune cells through CIBERSORT (related to Figure 7G).

**Supplementary Table 12.** The differential expression of immunotherapy response biomarkers in the high-risk and low-risk LUAD patients (related to Figure 7H).
